# Cleaning the operating theatre

**Published:** 2021-07-20

**Authors:** Ciku Mathenge, Yadav Ganesh Prasad

**Affiliations:** 1 Professor of Ophthalmology: University of Rwanda and Director of Training and Research: Rwanda International Institute of Ophthalmology, Rwanda.; 2 Senior Manager (Operation Theatre): Dr Agarwal’s Group of Eye Hospitals, Rwanda.

**Cleaning the operating theatre is an essential part of keeping patients and staff members safe. Here is how.**

Cleaning the operating theatre and its immediate environment minimises patients’ and health care workers’ exposure to potentially infectious microorganisms.

Cleaning happens at various times:

- When preparing a new operating theatre
- Every day, before surgery begins
- Between patients
- After the last operation of the day (known as terminal cleaning)
- Deeper cleans are carried out once a week and/or once a month.

All areas must be cleaned: unrestricted, semi-restricted and restricted areas. Start in the operating theatre before moving to the scrub areas, anaesthetic and recovery rooms, and then the sterilising area. The toilet should be cleaned last.

## Equipment

Cleaning equipment must be in plentiful supply. These should be a set for each for the operating theatre, the toilet, and ancillary rooms. Sets should be stored separately. Each set should contain the following:

- Mops and buckets
- Hard scrubbing brush
- Rubber pusher to remove excess water
- Disinfectant. Select disinfecting solutions with broad-spectrum microbicidal activity, which are appropriate for use on each type of surface that must be disinfected. Check with the pharmacy department what is available and use the best quality at an affordable price
- Absorbent dry and wet cleaning cloths
- Oil for lubricating equipment wheels.

The person doing the cleaning should change into a clean gown, cap, mask, and clean utility gloves.

**Cleaning tip**

**Have one mop for the clean rooms and operating theatre and a separate mop for the dirty areas. Change the water between each area.**

It is good practice to place a cautionary ‘Wet Floor’ sign at the entrance of the room when cleaning starts.

## Daily cleaning and disinfection

### Before the day’s surgery begins

Clean and disinfect the operating theatre every morning, irrespective of whether it will be used or not. Use warm, soapy water to clean, then wipe with a cloth soaked in clean water to move any soap (or detergent)residue. Finally, wipe with the disinfectant solution.

**Figure:**
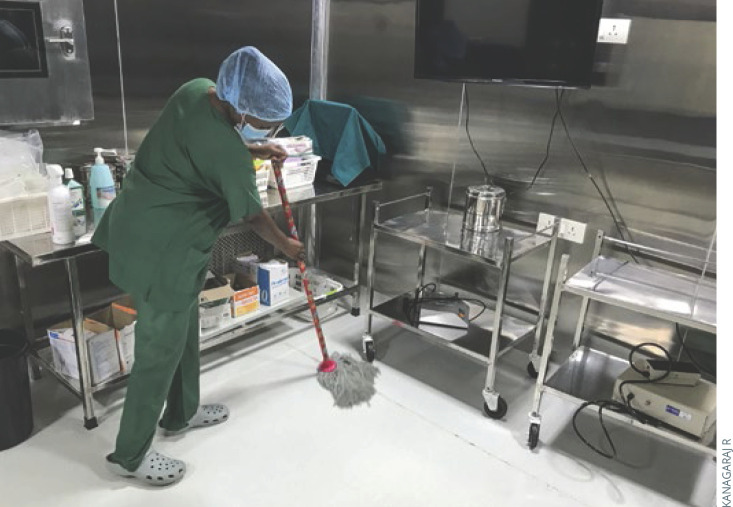
Dedicated cleaning equipment is used to clean the surgical area. **RWANDA**

Clean and disinfect the following:

- All flat surfaces (wipe from top to bottom, then from the centre outwards).
- The patient bed and its attachments, positioning devices, and patient transfer devices.
- Containers for sterile instruments, antiseptic bottles, and the trays in which these are kept.
- Scrub basins, taps, and walls. Check for any leaks.
- The soap and antiseptic solution bottles at the scrub basin (check that they are full and refill them if needed – this can be done the evening before if the operating theatre is temperature controlled).

Prepare waste bins by inserting colour-coded waste collection bags.

Finally, clean and disinfect the floor. Remove excess dirt and dust using either a mop or a hospital-grade wet vacuum, mop with clean water to remove soap residue, then mop using disinfectant. Take care not to agitate the dust, which spreads it.

Once the operating theatre is cleaned and disinfected, keep the door closed for 10–15 minutes with ventilation equipment turned on.

**Figure:**
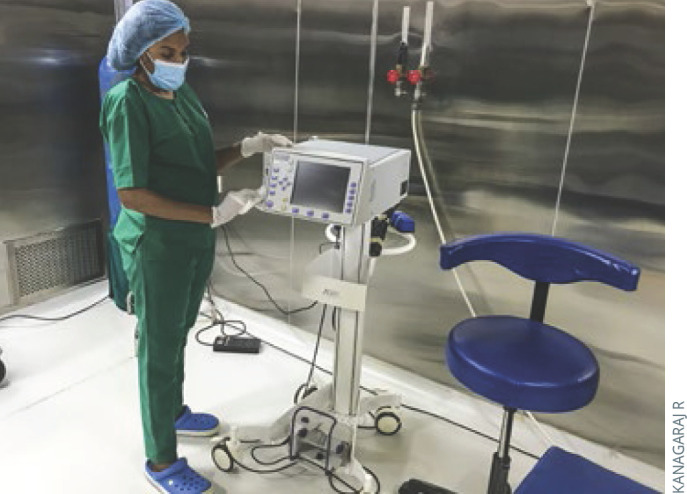
Clean all electronic equipment (e.g., phaco machines) according to the manufacturer’s instructions. **RWANDA**

### In between each patient

- After each operation, clean and disinfect any soiled areas of the floor as described previously.
- Clean and disinfect any furniture or equipment that came in contact with the patient or may have become soiled or damp, including the operating table, surgical lights, blood pressure cuffs, and tourniquets.
- Clean and disinfect the floor around the operating table (up to 1.5 metres away from the table).
- Collect and remove waste from the kick bucket and remove all other waste; replace all bin liners.
- Remove waste from equipment such as suction machines and clean, disinfect, or sterilise them, as appropriate.

### At the end of the day, after surgery

- Use a cloth and hot soapy water to wash all surfaces, including the tops of operating tables and all stools.
- Switch equipment off at the mains. Wipe down electrical cables carefully using a cloth dampened with a small amount of alcohol or other disinfectant (to ensure minimal usage of fluid).
- Clean the legs and wheels of trolleys and tables.
- Damp dust hanging lights and other items on the ceiling.
- Clean operating microscopes and operating lenses after each theatre session. Do not clean microscopes or lenses using soapy water, as soap residue can damage the lens. Use a soft, non-abrasive cloth for the lens and a cloth dampened with 70% alcohol or disinfectant for the microscope (as per the manufacturer’s instructions), including the handles.
- Clean anaesthesia machines and carts, IV poles, and patient monitors.
- Change hand towels, patient sheets, and blankets in the theatre and toilet area.
- Clean the floor.

### Other areas

- The toilets and changing rooms must be checked and cleaned throughout the day and separate cleaning equipment must be used for the toilets.
- Tea rooms and kitchens (and the recovery area, if food is given) must be cleaned and all leftover food and crumbs must be removed so that insects are not attracted to the area.

## Weekly cleaning

- Remove all articles from shelves and clean all surfaces thoroughly using hot, soapy water. **Note:** Do not get sterile items wet, as this will make the packaging permeable and the items will therefore no longer be sterile.
- Wash the floor and apply disinfectant.
- Wash and dry instrument trolleys, including the wheels and the rungs.
- Scrub bowls and gallipots.
- Clean windows inside and outside.
- Wipe all high surfaces, such as the tops of cupboards and windowsills, with a damp cloth. This is to prevent the build-up of dust.

## Monthly cleaning

- Move furniture such as cupboards or shelves away from the walls and clean the areas behind and underneath them. Clean the tops and the inside of cupboards, drawers and lockers. To prevent damage, remove all articles when doing so.
- Check expiry dates and rotate stock so that items with expiry dates in the near future are at the front.
- Clean trolleys, IV stands, stools, microscopes, etc., if needed.
- Wash curtains, if used in recovery areas, at least every three months.

**Figure:**
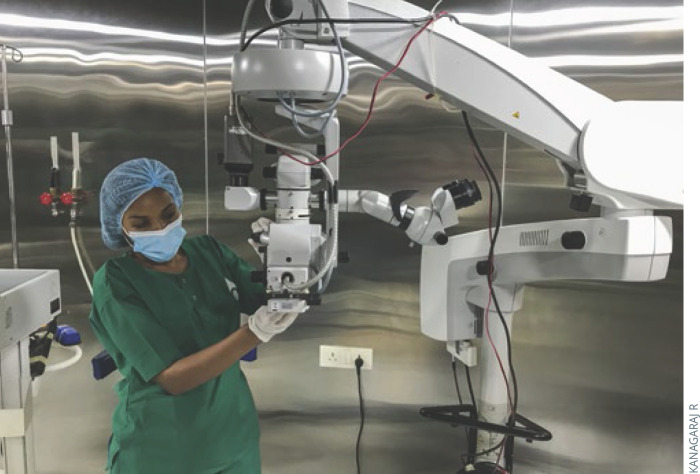
Clean the microscope knobs, nosepiece, levels, control rods, microscope stand and other areas of the device regularly with a damp cloth (not wet). Add a small amount of soap to help remove stubborn dirt and oil. **RWANDA**

## Other considerations

- **Pest control**. Liaise with the administrator and the maintenance team to schedule regular pest inspections and/or control measures to prevent pests. Any control measures need to be carried out when the operating theatre is not in use. For example, schedule this to take place a day before the weekend or a festival. This allows time for the operating theatre to settle and for any issues to be rectified before it is needed again.
- **Air conditioning units and filters**. Ensure these are checked and cleaned. Change the filters as required.

**Cleaning tip**

Avoid using and storing aerosol cans in the operating theatre as these are flammable and release particles into the air.

**The ‘three-bucket’ system**

**Figure:**
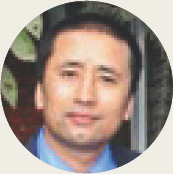
Kabindra Bajracharya Paediatric Ophthalmologist: Lumbini Eye Institute, Lumbini, Nepal.

Disinfectants can be inactivated by the presence of any dirt or biological material (known as ‘soil’) on a surface. They may also interact with soap or detergent residues. For optimal results, it is important that a surface is visibly clean before applying disinfectant.

1. First, mop the floor (or wipe the surface) using a detergent and water solution (in bucket 1) to get rid of any dirt or grime.
2. Second, mop the floor (or wipe the surface) using plain water (in bucket 2) to remove soap residue.
3. Third, once the floor is dry, mop it using disinfectant solution (in bucket 3); for example, 1% sodium hypochlorite solution.

When mopping, start from the corner of the room and work towards the door. Back away from the cleaned area.

